# Obesity Class Impacts Adverse Maternal and Neonatal Outcomes Independent of Diabetes

**DOI:** 10.3389/fendo.2022.832678

**Published:** 2022-03-24

**Authors:** Kirsten Neal, Shahid Ullah, Sarah J. Glastras

**Affiliations:** ^1^ Department of Medicine, Central Australian Health Service, Alice Springs, NT, Australia; ^2^ Department of Diabetes, Endocrinology and Metabolism, Royal North Shore Hospital, St Leonards, NSW, Australia; ^3^ College of Medicine and Public Health, Flinders University, Adelaide, SA, Australia; ^4^ Kolling Institute, Sydney, NSW, Australia; ^5^ Sydney Medical School, University of Sydney, Sydney, NSW, Australia

**Keywords:** large for gestational age (LGA), small for gestation age (SGA), preeclampsia, type 1 diabetes, type 2 diabetes, Caesarean, gestational diabetes, obesity

## Abstract

**Introduction:**

Obesity in pregnancy is a known risk factor for adverse maternal and neonatal outcomes. Few studies have compared adverse pregnancy-related outcomes according to obesity severity. Hence, we aimed to examine the impact of obesity class on maternal and perinatal outcomes.

**Methods:**

We retrospectively analysed data from all singleton births from mothers with obesity from 2013-2017 in Northern Sydney Local Health District in Sydney, Australia. Women were categorised into obesity class I (BMI 30-34.9kg/m2), class II (BMI 35-39.9 kg/m2) or class III (BMI 40+ kg/m2). Across BMI classes, we compared maternal outcomes including mode of delivery, gestational diabetes mellitus (GDM), and preeclampsia, and neonatal outcomes including large- and small-for-gestational age (SGA, LGA), neonatal hypoglycaemia, birth defects and timing of birth. Logistic analyses were performed to explore the impact of maternal obesity class on these outcomes, adjusting for maternal age, country of birth, parity, diabetes (both pre-existing and gestational) and hypertension.

**Results:**

There were 2466 births to women with obesity, class (69.1%), class II (21.8%), and class III (9.2%). 42.5% delivered by Caesarean section, 22.3% developed GDM and 11.2% had a hypertensive disorder in pregnancy, and Caesarean section and GDM were more common in women with higher class obesity. LGA occurred in 27.3% and SGA occurred in 4.0% of women across all classes of obesity. LGA rates were 49% more likely in women with class III compared to women with class I obesity (OR=1.49, CI 1.06-2.09, p=0.02). The presence of diabetes in the index pregnancy did not significantly impact risk of neonatal LGA between maternal obesity classes. Other neonatal adverse outcomes such as stillbirth and birth defects were more common in women with higher class obesity. SGA, neonatal hypoglycaemia, gestational age at delivery, APGAR 5-minute score and NICU admissions were similar across obesity classes, after adjustment for covariates.

**Conclusions:**

Obesity class increases the risk of many adverse maternal and neonatal outcomes. Obesity class is independently associated with LGA incidence in the neonate, independent of maternal factors including GDM. Ongoing efforts must be made to reduce obesity incidence in women of reproductive age to circumvent the adverse perinatal outcomes associated with obesity.

## Introduction

Obesity is a global health problem and its prevalence in pregnancy is increasing, particularly in Western countries (1, 2). In Australia, more than 1 in 5 women have obesity at the time of conception (3). Obesity in pregnancy increases maternal, neonatal and childhood risk of adverse outcomes. In pregnancy, maternal obesity increases the risk of gestational diabetes mellitus, gestational hypertension, pre-eclampsia, instrumental delivery, Caesarean section delivery and stillbirth (1, 2, 4, 5). Women with obesity are more likely to deliver a neonate with congenital abnormalities, large-for-gestational age (LGA) and respiratory distress syndrome (6–8).

Though many studies have established the association between maternal obesity and adverse perinatal outcomes, fewer studies have compared perinatal outcomes by obesity class. Obesity is defined as body mass index (BMI) ≥30kg/m^2^ and can be further stratified by class: class 1 (BMI 30.0 to 34.9 kg/m^2^), class II (BMI 35.0 to 39.9 kg/m^2^), and class III and above (BMI ≥40 kg/m^2^) (9). A large retrospective study found that increasing class of obesity was associated with increased rates of gestational hypertension, gestational diabetes, shoulder dystocia, Caesarean section, LGA, neonatal metabolic abnormalities, neonatal intensive care unit (NICU) admission and stillbirth (10). However, this study compared women with extreme obesity with the general pregnant population, without knowledge of the BMI status of the comparison group. A large UK-based study observed that women within higher BMI classes were more likely to have post-term birth as well as preterm birth (11). There is currently an evidence gap of the impact of varying classes of obesity on pregnancy-related outcomes, particularly how they interact with diabetes and other maternal risk factors for adverse outcomes.

Given obesity in pregnancy has become so common in many countries across the globe, our study objective was to understand the impact of obesity class on adverse perinatal outcomes within a cohort of women with obesity, specifically investigating the impact of obesity class on important maternal and neonatal outcomes, including LGA. We aimed to determine the relationship between obesity class and adverse outcomes, specifically adjusting for known comorbidities likely to be encountered by women with obesity, including gestational diabetes and hypertensive disorders. We hypothesised that increasing class of obesity is associated with increased risk of maternal and neonatal adverse outcomes.

## Materials and Methods

### Study Population

This retrospective cohort study identified pregnant women with a BMI >30kg/m^2^ recorded at their first antenatal visit, from within the ObstetriX and eMaternity databases from January 2013 to December 2017, within the Northern Sydney Local Health District in Sydney, NSW, Australia. These databases are routinely used in NSW and capture all antenatal and pregnancy outcomes for non-Indigenous women. Women aged 18 years and older with a singleton pregnancy, gestation 22 to 42 weeks were included in the cohort, and women who had a multiple pregnancy were excluded. Ethics approval was obtained from the NSLHD Human Research Ethics Committee. Ethnicity data was recorded based on country of birth; “Anglosphere” included Australia, New Zealand, Canada, USA and UK, “Asia” included all Asian and subcontinental countries, “other” were all other countries of birth.

### Exposure and Outcome Measures

Baseline maternal characteristics including age, country of birth, previous term pregnancy, pre-existing diabetes including gestational diabetes, pre-existing hypertension, current gestational diabetes or hypertension were recorded. Women with obesity were categorized into three groups according to the severity of obesity based on the World Health Organization classification system: obesity class I (BMI 30-34.9 kg/m^2^), obesity class II (BMI 35-39.9 kg/m^2^) and obesity class III and above (BMI 40+ kg/m^2^) (9). Maternal outcomes of interest included mode of delivery (normal vaginal delivery, instrumental vaginal delivery, Caesarean section), gestational diabetes and hypertensive disorders (essential, gestational and pre-eclampsia). Neonatal outcomes included live birth and stillbirth, birth weight, LGA, small-for-gestational age (SGA), gestational age at delivery, APGAR 5-minute score, hypoglycaemia, birth defect, birth injury, respiratory distress and NICU admission at birth. LGA was defined as birthweight > 90^th^ centile for gestational age, and SGA defined as birthweight < 10^th^ centile for gestational age, based on the WHO weight percentiles calculator. Timing of birth was divided into preterm <37 weeks, early term 37 – 37.9 weeks, and term >38 weeks) with gestational age being represented as week and gestational day throughout the study (e.g., 39.5 represented 39 weeks and 5/7 days).

### Statistical Analysis

ANOVA was used to explore the significance of differences in baseline characteristics between the three classes of obesity. The standard Chi-square test for association with continuity correction, was utilised. The normality assumption was checked visually by frequency histogram and normal Q-Q plot and analytically by Anderson-Darling test. Median and Interquartile range (IQR) were calculated for skewed data and compared by standard Kruskal Wallis test. A logistic regression models were applied to examine the neo-natal and maternal outcomes between the obesity groups. For each outcome of interest, univariate models were first performed without adjustment. Then, multivariate analysis was undertaken, adjusting for covariates (baseline characteristics and presence of gestational diabetes or hypertensive disorders in the index pregnancy). The estimates were calculated using the maximum likelihood method and were expressed as odds ratios (ORs), and statistical significance defined by the 95% CI>1.0. The interaction of variables was utilized to determine interaction between obesity class and pre-existing or pregnancy-related diabetes in determining LGA, statistical significance P<0.05. Model diagnostics and goodness of fit were evaluated by Receiver Operator Characteristic (ROC) curves. The two-sided test was performed for all analysis and the level of significance was set at p < 0.05. All statistical analyses were performed using Stata statistical software, version 16.1.

## Results

A total of 2466 births in women with obesity were recorded between 2013 and 2017 within the NSLHD. The demographic and clinical characteristics of the population by obesity class are shown in **Table 1**. 1703 women (69.1%) had obesity class I, 537 women (21.8%) had class II, and 226 women (9.2%) had class III or above obesity. The maternal age of the participants ranged between 18 to 45 years old, mean 32.3±5.1years. Most women in this study were born in Anglosphere countries (74.6%) and there was a trend for women from Anglosphere countries being categorized in higher BMI classes than women from Asian or other backgrounds (P<0.01). Most women within this study cohort had a previous term or nonviable pregnancy (78% and 75.5% respectively), and there was a trend for greater number of term pregnancies in women with higher class obesity (P<0.01). 4.8% of women within the cohort had a previous history of gestational diabetes and 1.7% had pre-existing type 1 or type 2 diabetes. Women with higher BMI class were more likely to have had previous gestational diabetes (4.3%, 5.0% and 8.4% in obesity class I, II and III and above respectively, P<0.02), and a history of essential hypertension (1.8%, 2.4% and 4.9% in obesity class I, II and III and above respectively, P<0.001).

**Table 1:** Women’s socio-demographic and clinical characteristics by obesity class.

Characteristics	All n (%)	BMI obesity group, n (%)			P value
		Class I obesity (30-34.9)	Class II obesity (35-39.9)	Class III obesity (40+)	
N	2466	1703 (69.1)	537 (21.8)	226 (9.2)	
Age, y, mean (SD)	32.3 (5.1)	32.3 (5.1)	32.0 (4.9)	32.7 (5.1)	0.17
Age, y					0.23
<25	160 (6.5)	117 (6.9)	31 (5.8)	12 (5.3)	
25-29	566 (23.0)	383 (22.5)	133 (24.8)	50 (22.1)	
30-34	918 (37.2)	616 (36.2)	218 (40.6)	84 (37.2)	
35-39	628 (25.5)	455 (26.7)	116 (21.6)	57 (25.2)	
40+	194 (7.9)	132 (7.8)	39 (7.3)	23 (10.2)	
Country of birth					<0.001
Anglosphere	1839 (74.6)	1211 (71.1)	440 (81.9)	188 (83.2)	
Asian	339 (13.7)	289 (17.0)	39 (7.3)	11 (4.9)	
Others	288 (11.7)	203 (11.9)	58 (10.8)	27 (11.9)	
Previous Term pregnancy				<0.01
0	542 (22.0)	375 (22.0)	115 (21.4)	52 (23.0)	
1	893 (36.2)	635 (37.3)	193 (35.9)	65 (28.8)	
2	294 (11.9)	195 (11.5)	66 (12.3)	33 (14.6)	
3+	160 (6.5)	93 (5.5)	42 (7.8)	25 (11.1)	
Missing	577 (23.4)	405 (23.8)	121 (22.5)	51 (22.6)	
Nonviable pregnancy					0.60
0	604 (24.5)	427 (25.1)	127 (23.6)	50 (22.1)	
1	506 (20.5)	341 (20.0)	113 (21.0)	52 (23.0)	
2	193 (7.8)	135 (7.9)	41 (7.6)	17 (7.5)	
3+	124 (5.0)	78 (4.6)	30 (5.6)	16 (7.1)	
Missing	1039 (42.1)	722 (42.4)	226 (42.1)	91 (40.3)	
History of previous gestational diabetes				0.02
No	2334 (94.6)	1622 (95.2)	508 (94.6)	204 (90.3)	
Yes	119 (4.8)	73 (4.3)	27 (5.0)	19 (8.4)	
Missing	13 (0.5)	8 (0.5)	2 (0.4)	3 (1.3)	
Pre-existing type I/II diabetes			0.39
No	2410 (97.7)	1669 (98.0)	524 (97.6)	217 (96.0)	
Yes	43 (1.7)	26 (1.5)	11 (2.0)	6 (2.7)	
Missing	13 (0.5)	8 (0.5)	2 (0.4)	3 (1.3)	

Data is presented as N (%) unless stated otherwise, SD, Standard deviation. Anglosphere was defined as country of birth including Australia, New Zealand, USA, UK, Canada, Asian was defined as country of birth in all central, subcontinent Asian countries, and other included all other countries of birth.

### Maternal Outcomes

**Table 2** shows the adverse maternal outcomes across BMI classes. All adverse maternal outcomes were higher as BMI class increased. Overall, 42.5% of women with obesity delivered by Caesarean section, 22.3% developed gestational diabetes and 11.2% had a hypertensive disorder during pregnancy. Women with higher BMI class had significantly higher rates of Caesarean delivery, gestational diabetes and hypertensive disorders. Of note, 6.2% of women with class III and above obesity developed pre-eclampsia.

**Table 2:** Maternal outcomes by obesity class.

Maternal outcomes	All n (%)	BMI obesity group, n (%)			P value
		Class I obesity (30-34.9)	Class II obesity (35-39.9)	Class III obesity (40+)	
N	2466	1703 (69.1)	537 (21.8)	226 (9.2)	
Mode of birth					<0.001
NVD	1182 (47.9)	842 (49.4)	254 (47.3)	86 (38.1)	
Instrumental VD	235 (9.5)	177 (10.4)	39 (7.3)	19 (8.4)	
LSCS	1048 (42.5)	683 (40.1)	244 (45.4)	121 (53.5)	
Missing	1 (<1)	1 (0.1)	–	–	
Current gestational diabetes					0.02
No	1858 (75.3)	1308 (76.8)	395 (73.6)	155 (68.6)	
Yes	550 (22.3)	357 (21.0)	130 (24.2)	63 (27.9)	
Missing	58 (2.4)	38 (2.2)	12 (2.2)	8 (3.6)	
Hypertension (HTN)					<0.001
No	2166 (87.8)	1525 (89.5)	460 (85.7)	181 (80.1)	
Essential HTN	54 (2.2)	30 (1.8)	13 (2.4)	11 (4.9)	
Gestational HTN	139 (5.6)	83 (4.9)	36 (6.7)	20 (8.8)	
Pre-eclampsia	85 (3.4)	48 (2.8)	23 (4.3)	14 (6.2)	
Missing	22 (0.9)	17 (1.0)	5 (0.9)	–	

**Figure 1:**
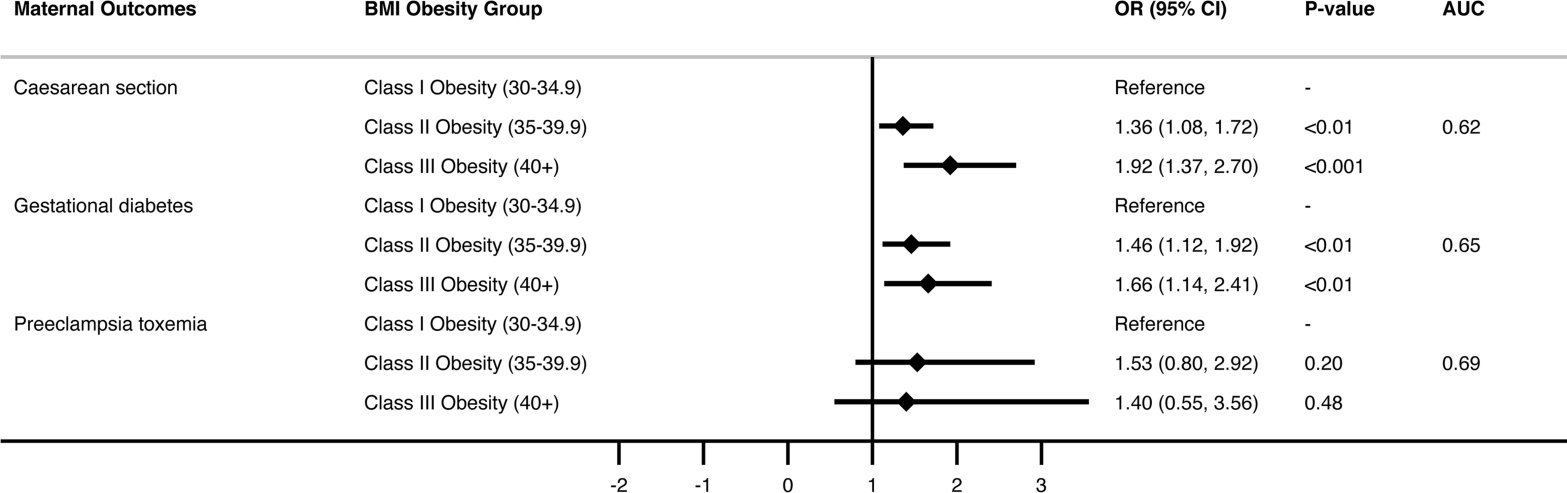
Multivariate logistic regression model of three maternal outcomes by BMI group.

Multivariate analysis (**Figure 1** and **Supplementary Table 1**) showed almost a two-fold increased risk of caesarean delivery with increasing BMI class, OR 1.92, p<0.001, for obesity class III and above compared with obesity class I. Risk of caesarean delivery was increased almost three-fold in those with pre-eclampsia [OR 2.94 (1.58-5.46), p< 0.01], two-fold when age > 40 compared with age < 25 [OR 2.10 (1.24-3.55), p<0.01], and risk was halved in women with 3 or more previous pregnancies compared with primigravida women [OR.53 (0.36 -0.78), p<0.001].

Multivariate analysis (**Figure 1** and **Supplementary Table 2**) showed approximately 1.5-fold increased risk of GDM with increasing obesity class (class II obesity OR 1.46, class III and above OR 1.66 compared with class I obesity, p<0.01). Risk of GDM was also significantly increased with increasing age (OR 4.95 for age >40 compared with age <25, p<0.01) and in Asian compared with Anglosphere born women (OR 3, p<0.001).

Pre-eclampsia occurred in only 85 women, but in the multivariate analysis, risk was not significantly increased across BMI categories after accounting for age, country of birth and presence of any diabetic disorder (**Figure 1** and **Supplementary Table 3**).

### Neonatal Outcomes

**Table 3** shows the adverse neonatal outcomes across BMI class. Despite small numbers of stillbirth (N = 7) there were significantly more stillbirths in women with higher class obesity (P<0.05). The mean birth weight was 3447g which was similar across BMI classes. There were no differences across BMI class for low or high birth weight outcomes. Mean gestational age at delivery was 39.3 weeks, and timing of delivery was earlier in women within higher BMI class (39.3 vs 39.1 weeks in obesity class I vs III, P<0.01). There was a trend towards earlier term deliveries in women with higher class obesity however this did not reach statistical significance (P= 0.08). 5-minute Apgar score less than 7 was uncommon across the study cohort (3.4%).

**Table 3:** Neonatal outcomes by obesity class.

Neonatal outcomes	All n (%)	BMI obesity group, n (%)			P value
		Class I obesity (30-34.9)	Class II obesity (35-39.9)	Class III obesity (40+)	
N	2466	1703 (69.1)	537 (21.8)	226 (9.2)	
Neonatal outcome					0.05
Live birth	2459 (99.7)	1701 (99.9)	534 (99.4)	224 (99.1)	
Still birth	7 (0.3)	2 (0.1)	3 (0.6)	2 (0.9)	
Birthweight, g, Mean (SD)	3447.2 (666.9)	3447.4 (653.0)	3445.4 (658.7)	3450.1 (783.4)	0.99
Birthweight, g					0.55
Low (<2500)	148 (6.0)	97 (5.7)	34 (6.3)	17 (7.5)	
>Normal (2500-3999)	1913 (77.6)	1327 (77.9)	420 (78.2)	166 (73.5)	
High (4000+)	405 (16.4)	279 (16.4)	83 (15.5)	43 (19.0)	
Gestational age, weeks, median (IQR)	39.3 (38.4-40.3)	39.3 (38.4-40.3)	39.2 (38.4-40.2)	39.1 (38.1-40.0)	<0.01
Gestational age, weeks					0.08
Term (38+)	2093 (84.9)	1460 (85.7)	453 (84.4)	180 (79.6)	
Early term (37-37.9)	169 (6.9)	109 (6.4)	35 (6.5)	25 (11.1)	
Pre-term (<37)	204 (8.3)	134 (7.9)	49 (9.1)	21 (9.3)	
APGAR 5m					0.18
7+	2377 (96.4)	1648 (96.8)	515 (95.9)	214 (94.7)	
<7	85 (3.4)	52 (3.1)	21 (3.9)	12 (5.3)	
Missing	4 (0.2)	3 (0.2)	1 (0.2)	–	
LGA					0.12
Normal size	1792 (72.7)	1254 (73.6)	386 (71.9)	152 (67.3)	
LGA	674 (27.3)	449 (26.4)	151 (28.1)	74 (32.7)	
SGA				0.50
Normal size	2368 (96.0)	1365 (96.0)	513 (95.5)	220 (97.3)	
SGA	98 (4.0)	68 (4.0)	24 (4.5)	6 (2.7)	
Hypoglycaemia				0.17
No	2278 (92.4)	1575 (92.5)	501 (93.3)	202 (89.4)	
Yes	188 (7.6)	128 (7.5)	36 (6.7)	24 (10.6)	
Birth defect					0.01
No	2401 (97.4)	1659 (97.4)	528 (98.3)	214 (94.7)	
Yes	63 (2.6)	42 (2.5)	9 (1.7)	12 (5.3)	
Missing	2 (0.1)	2 (0.1)	–	–	
Birth injury					0.54
No	2456 (99.6)	1696 (99.6)	534 (99.4)	226 (100.0)	
Yes	10 (0.4)	7 (0.4)	3 (0.6)	–	
Respiratory distress					0.27
No	2227 (90.3)	1541 (90.5)	477 (88.8)	209 (92.5)	
Yes	239 (9.7)	162 (9.5)	60 (11.2)	17 (7.5)	
NICU Admission at Birth					0.19
No	2103 (85.3)	1440 (84.6)	462 (86.0)	201 (88.9)	
Yes	363 (14.7)	263 (15.4)	75 (14.0)	25 (11.1)	

Data were presented as N (%) unless stated otherwise; SD, Standard deviation; NVD, Normal Vaginal Delivery; LSCS, lower (Uterine) Segment Caesarean Section.

LGA occurred in 27.3% and SGA occurred in 4.0% of women across all classes of obesity. Neonatal hypoglycaemia occurred in 7.6% of all neonates. There were low rates of birth defects (2.6%), and birth injury was uncommon. Respiratory distress was observed in 9.7% of neonates, and NICU admission occurred in 14.7% of neonates born to mothers with obesity in this cohort.

**Figure 2:**
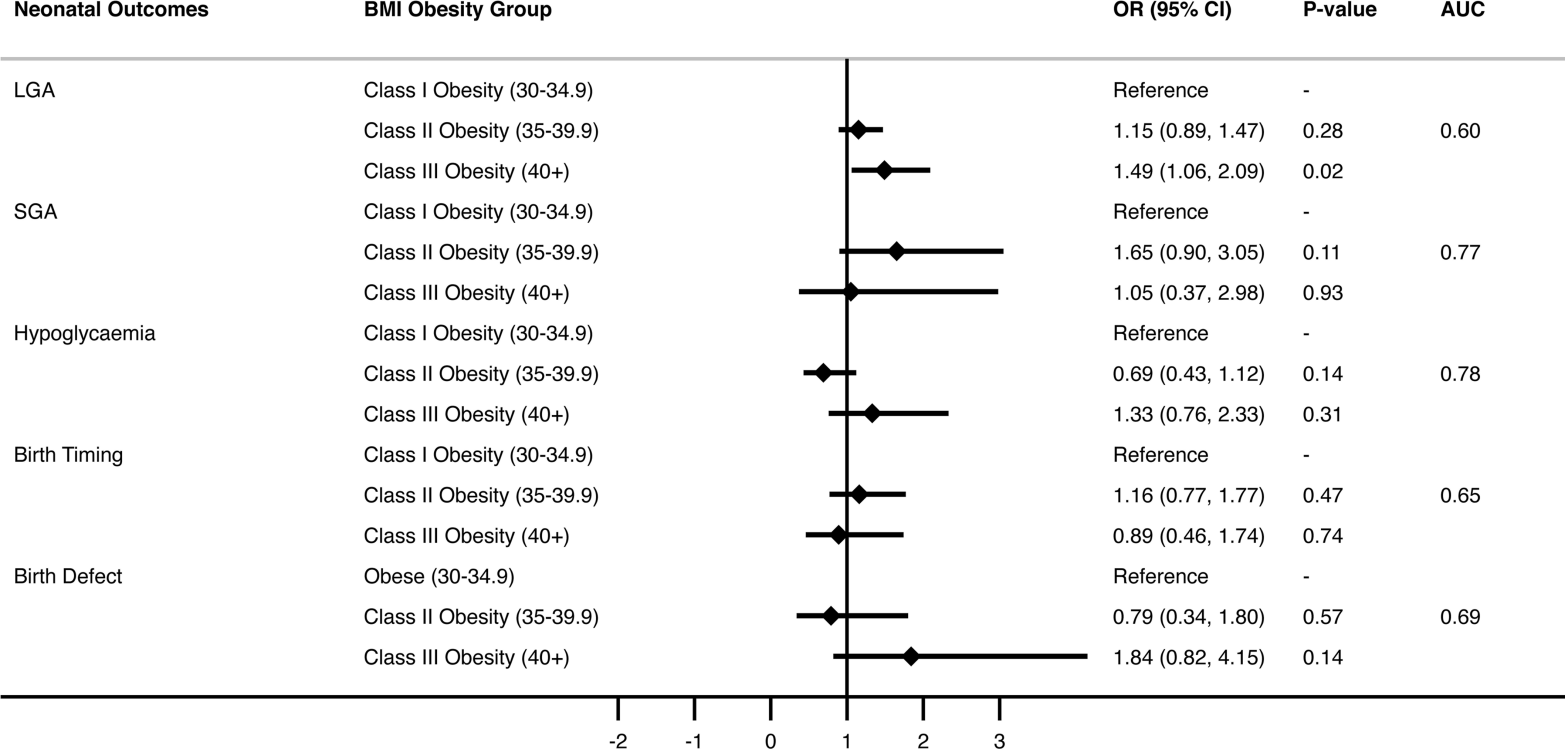
Multivariate logistic regression model of five neonatal outcomes by BMI group.

Multivariate analysis, adjusting for covariates including maternal age, country of birth, previous term pregnancy, pre-pregnancy diabetes and hypertension, and GDM or hypertension in the current pregnancy were performed (**Figure 2** and **Supplementary Table 4**). LGA rates were 49% more likely in women with obesity class III and above compared to women with class I (OR=1.49, CI 1.06-2.09, p=0.02). Further analysis of this association found that women with prior pregnancies had a significantly higher chance of delivering an LGA neonate compared to nulliparous women, exaggerated by increasing BMI class. Furthermore, women aged over 40 years had increased risk of LGA compared with women aged under 25 (OR=1.86, 95% CI 2.16-2.99, P=0.01), and women born in Asia had less risk of LGA neonate compared to the Anglosphere group (OR=0.57, 95% CI 0.41-0.81, P<0.01). Finally, women with pre-existing type 1 or 2 diabetes had a 2-fold increased risk of LGA across all BMI classes (OR=2.11, 95% CI 1.01-4.42, p=0.046). The presence of GDM in the index pregnancy did not significantly impact risk of neonatal LGA between maternal obesity classes. No significant interaction was found between the presence of diabetes (Type 1, 2 or GDM) and obesity classes for outcome of neonatal LGA (**Table 4**).

**Table 4:** Interaction between obesity class and diabetes status for LGA.

Comorbidities	OR (95% CI)		
	Class I Obesity (30-34.9)	Class II obesity (35-39.9)	Class III obesity (>=40)
LGA			
Diabetes status			
No	Reference	Reference	Reference
Pre-existing type I/II diabetes	1.70 (0.76-3.78)	0.96 (0.25-3.70)	4.20 (0.74-23.75)
Current gestational diabetes	0.82 (0.63-1.08)	1.02 (0.66-1.59)	0.98 (0.52-1.84)

After adjusting for covariates, obesity class did not impact SGA risk. However, women with pre-existing essential hypertension prior to pregnancy, current gestational hypertension or pre-eclampsia were at least 3 times more likely to deliver an SGA neonate (OR 3.66, 95% CI 0.99-13.5, p=0.05, OR 3.85, 95% CI 1.69 – 8.75, p < 0.01, OR 15.97, 95% CI 7.8-32.68, p < 0.001 respectively, **Figure 2** and **Supplementary Table 5**). Asian-born women had higher rates of SGA compared with “Anglosphere” born women (OR 3.72 95% CI 1.93 – 7.18, p <0.001).

There was no statistical difference in neonatal hypoglycaemia between obesity class in the unadjusted or adjusted models (**Figure 2** and **Supplementary Table 6**). Neonates of women with pre-existing type 1 or 2 diabetes were most likely to have hypoglycaemia (OR 24.95 95% CI 10.92-57.01, p< 0.001), and neonates of women with GDM in the index pregnancy were also at greater risk [OR 7.87 (5.30-11.69] p < 0.001). Asian-born women (compared with the “Anglosphere” women) and those > 35 years old (compared to < 25 years old) were also at increased risk of neonatal hypoglycaemia.

BMI class did not have a statistically significant impact on timing of delivery in either univariate or multivariate analysis (**Figure 2** and **Supplementary Table 7**). The most striking association between maternal characteristic and birth timing was pre-existing type 1 or type 2 diabetes that increased the risk of earlier delivery by 3-fold (OR 2.81 in unadjusted model and OR 3.2 in adjusted model). Furthermore, preeclampsia increased the risk of early delivery by 9-fold (OR 9.48 and 9.04, P<0.001 respectively).

Birth defects were present in 63 neonates and in the unadjusted model were two-fold more likely in women with obesity class III and above compared with class I (OR 2.21, 95% CI 1.15-4.27, p=0.02; **Figure 2** and **Supplementary Table 8**). After adjusting for covariates, there was no longer statistical significance. There was no association between maternal age and birth defects. Previous term delivery conferred reduced risk of birth defects in both the unadjusted and adjusted models. The presence of diabetes or hypertension was not associated with birth defects in this cohort.

## Discussion

This retrospective study found that greater class of obesity has important implications for adverse maternal and neonatal outcomes in a cohort of women with obesity. Women with obesity class III and above have increased rates of caesarean section, gestational diabetes and hypertensive disorders (essential and gestational hypertension and pre-eclampsia). Though a small effect size, stillbirth incidence was greater in women with higher obesity class. The most striking neonatal risk associated with higher class of obesity was LGA, even after adjustment for confounding variables including diabetes in any form. Women with obesity class III and above had a 49% increased risk of LGA. Our study did not show increased incidence of SGA in neonates born to women with higher classes of obesity.

Despite guidelines recommending that women with obesity, including BMI >40kg/m^2^, be counselled to birth *via* normal vaginal delivery at 40 weeks gestation, our study showed that women with class III obesity and above are most likely to deliver *via* Caesarean section (53.5%) and prior to 40 weeks (close to 39 weeks) (12). Because our study was population-level data, we were unable to ascertain whether Caesarean section was spontaneous or elective. Compared with the Australian Caesarean rate of 33% [2018 AIHW statistics (13)], women with obesity had a much higher rate of Caesarean section, 42.5% in our obese cohort, up to 53.5% in obesity class III and above. The fact that the higher obesity classes had an almost two-fold increased risk of caesarean delivery compared with class I obesity, even after accounting for associated risk factors including age, hypertensive and diabetic disorders, suggests obesity itself is an independent risk factor for caesarean delivery.

There was a significant association between obesity class and pre-eclampsia, such that women with obesity class III and above had at least a two-fold increased risk of pre-eclampsia compared to women with class I obesity. The incidence of pre-eclampsia in women with obesity was 3.4% in our study, increasing to 6.2% in the women with obesity class III and above (p< 0.01). It seems the difference in risk seen across BMI categories was accounted for by comorbidities associated with obesity (age, hypertensive and diabetic disorders, multiparity and country of birth) and not due to obesity itself. This is despite recommendations for women with BMI > 35 to be placed on aspirin for preeclampsia prevention (12). It is important to note that rates of pre-eclampsia were fairly low in our cohort (N=85), possibly contributing to the lack of statistical significance in the multivariate analysis. The association between increasing BMI and pre-eclampsia is well appreciated and clearly documented in several systematic reviews and meta-analyses (14, 15). Pre-eclampsia itself was found to be the most significant risk factor for delivering a small baby, increasing the risk by fifteen-fold.

Birth weight is an important determinant of neonatal complications soon after birth, such as respiratory distress syndrome, hyperbilirubinaemia and neonatal hypoglycaemia, and LGA size at birth is associated with long-term health outcomes, including obesity, type 2 diabetes and cardiovascular disease (16–19). The results of this study demonstrated a 49% increased risk of LGA with greater class of obesity. This finding is consistent with current literature which demonstrates a linear relationship between increasing maternal body weight and birth weight (20–22). The presence of pre-existing diabetes (type 1 or 2) more than doubled the risk for developing LGA, however pre-existing diabetes did not amplify the impact of obesity class on risk of LGA.

In this study, after adjusting for known risk factors for SGA such as advanced maternal age and hypertensive disorders, we found no significant impact of obesity class on SGA. Previous studies have suggested mixed reports on women with obesity and rates of SGA compared to normal-weight women. A large study performed in China found that 17.6% of women with obesity gave birth to an SGA-offspring compared to 7.4% of normal-weight women. In our study, the rate of SGA was much lower at 4%, which is comparable to SGA rates reported in women with obesity within a Scandinavian cohort (23). Similarly, in this Scandinavian study SGA rates were not increased with increasing class of obesity. Therefore, though obesity is a known risk factor for SGA generally, our study concretises the evidence that class of obesity continues to affect LGA rates but has no further impact on SGA incidence.

Our study confirms that neonatal hypoglycaemia is not greater in women with higher obesity class, and our data confirms diabetes is the major determinant of neonatal hypoglycaemia, the incidence of which was lower in women with higher obesity class in our cohort. Our study showed that women with class III obesity and above were more likely to deliver a neonate with a birth defect, however this effect was no longer significant after adjusting for other known risk factors for birth defects, including diabetes and advanced maternal age. Timing of birth was earlier in women with more severe obesity however after adjustment for covariates, the effect was no longer significant. Our study showed a small significant difference in timing of delivery (39.3 vs 39.1 weeks in obesity class 1 vs class III and above). This likely reflects obstetric decisions regarding timing of delivery; frequently women with BMI > 40kg/m2, those with suspected LGA and those with diabetes on medication (gestational diabetes or pre-existing) are delivered at 38 – 39 weeks. Women with class III obesity and above are more likely to be managed by tertiary level specialist care, pregnancies are more closely monitored, and carefully managed with a greater likelihood of delivering within the 38^th^ week. The earlier gestation may also be attributable to earlier intervention due to higher rates of comorbid conditions such as pre-eclampsia, hypertensive disorders, and diabetes.

Within our cohort, there were very few stillbirths (N=7), however the incidence was statistically greater in women in the higher BMI class (obesity class I 0.1%, class III and above 0.9%, P=0.05). Compared to the Australian average of 2.2 in 1000 stillbirths, these numbers are proportionally high (24). However, compared to the global statistics of 13.9 in 1000 stillbirths, the number of stillbirths in our group of women with obese class III and above is low, which highlights the importance of good obstetric care and management (24). Our dataset did not include information about assisted reproductive technology (e.g., IVF) versus spontaneous conception, an important limitation given the known association between assisted reproductive technologies and adverse perinatal outcomes, such as stillbirth (25).

Our study population was predominantly Caucasian. Nonetheless, our data showed that neonates born to Asian women were less likely to have LGA and more likely to have SGA than their Anglosphere counterparts. It is well-established that babies born to women from Asian ethnicity are smaller in size than Caucasian babies (26, 27). A limitation of this study is that women and their neonates were classified by body weight against the WHO classification system, which does not take into account Asian-specific differences in BMI (27). A further limitation of our study is that women were categorised by country of birth, rather than ethnicity, assuming that the country of birth correlated with ethnicity. However, given the demographics of our population were predominantly Caucasian, and that Asian women residing in northern Sydney are mostly first or second generation Asian Australian-born (10 to 25% based on 2016 ABS census results), we likely underestimate rather than overestimate LGA in this population.

Our study could not assess the impact of excessive gestational weight gain, a factor known to be associated with adverse perinatal outcomes, including incidence of LGA (28). In our study, we did find that women with obesity class III and above were more likely to be multiparous; 14% of women in obesity class III and above had more than 3 term pregnancies. It is well documented that multiparous women are more likely to have obesity in a subsequent pregnancy (29). Meta-analysis of 17 studies concluded parity was associated with higher pre‐pregnancy BMI (30). Given the observed adverse maternal and perinatal outcomes associated with higher BMI class in our study, together with a large body of evidence, inter-pregnancy weight management strategies should be a major public health focus to reduce obesity and obesity class leading into pregnancy. A large Swiss study of over 300,000 singleton pregnancies showed that adverse pregnancy outcomes in women with obesity who lost weight between pregnancies from obese to non-obese class led to a 6.3% risk reduction of delivering a large baby in a subsequent pregnancy (31). This reinforces the importance of strong public health messages to prevent obesity in women of reproductive age.

In conclusion, obesity class has a significant impact on pregnancy-related complications, including maternal hypertensive disorders, diabetes disorders and Caesarean section. Neonates born to women with greater obesity class have significantly greater rates of LGA. The lack of significant difference seen in many of the adverse neonatal events across the BMI groups in this cohort highlights the importance of good obstetric care to manage the comorbidities associated with obesity in pregnancy. Further work in pre-conception weight management should be considered to reduce the number of women entering pregnancy with higher class of obesity.

## Data Availability Statement

The raw data supporting the conclusions of this article will be made available by the authors, without undue reservation.

## Ethics Statement

The studies involving human participants were reviewed and approved by NSLHD Human Research Ethics Committee. Written informed consent for participation was not required for this study in accordance with the national legislation and the institutional requirements.

## Author Contributions

KN drafted manuscript and performed preliminary analyses. SU performed statistical analyses. SG designed the study, edited and reviewed manuscript. All authors contributed to the article and approved the submitted version.

## Funding

Funding for this project was provided by in kind support from the Department of Endocrinology, Royal North Shore Hospital.

## Conflict of Interest

The authors declare that the research was conducted in the absence of any commercial or financial relationships that could be construed as a potential conflict of interest.

## Publisher’s Note

All claims expressed in this article are solely those of the authors and do not necessarily represent those of their affiliated organizations, or those of the publisher, the editors and the reviewers. Any product that may be evaluated in this article, or claim that may be made by its manufacturer, is not guaranteed or endorsed by the publisher.
