# Supplementary material for: Obesity Class Impacts Adverse Maternal and Neonatal Outcomes Independent of Diabetes
Source: Front Endocrinol (Lausanne). 2022 Mar 24;13:832678. doi: 10.3389/fendo.2022.832678 (PMC8987983; doi:10.3389/fendo.2022.832678)
Supplement: Supplementary file 1 [file DataSheet_1.docx]

# SUPPLEMENTARY MATERIAL

## Supplementary Table 1: Univariate and multivariate logistic regression model of LGA by obesity class

| Characteristics | | | Univariate model | | Multivariate model | |
| --- | --- | --- | --- | --- | --- | --- |
|  | | | OR (95% CI) | P Value | OR (95% CI) | P Value |
| BMI Obesity group | | |  |  |  |  |
|  | Class I Obesity (30-34.9) | | Reference | - | Reference | - |
|  | Class II obesity (35-39.9) | | 1.09 (0.88-1.36) | 0.42 | 1.15 (0.89-1.47) | 0.28 |
|  | Class III obesity (>=40) | | 1.36 (1.01-1.83) | 0.04 | 1.49 (1.06-2.09) | 0.02 |
| Age, y | | |  |  |  |  |
|  | <25 | | Reference | - | Reference | - |
|  | 25-29 | | 1.32 (0.87-2.00) | 0.19 | 1.58 (0.92-2.73) | 0.10 |
|  | 30-34 | | 1.27 (0.85-1.89) | 0.24 | 1.27 (0.75-2.16) | 0.37 |
|  | 35-39 | | 1.24 (0.82-1.87) | 0.31 | 1.23 (0.72-2.11) | 0.44 |
|  | 40+ | | 1.86 (1.16-2.99) | 0.01 | 1.87 (1.03-3.39) | 0.04 |
| Country of birth | | |  |  |  |  |
|  | | Anglosphere | Reference | - | Reference | - |
|  | | Asian | 0.51 (0.38-0.68) | <0.001 | 0.57 (0.41-0.81) | <0.01 |
|  | | Other | 0.85 (0.64-1.22) | 0.25 | 0.84 (0.61-1.15) | 0.28 |
| Term pregnancy | | |  |  |  |  |
|  | 0 | | Reference | - | Reference | - |
|  | 1 | | 1.52 (1.19-1.95) | <0.01 | 1.63 (1.27-2.09) | <0.001 |
|  | 2 | | 1.68 (1.22-2.30) | <0.01 | 1.70 (1.23-2.35) | <0.01 |
|  | 3+ | | 1.78 (1.22-2.62) | <0.01 | 1.63 (1.09 -2.42) | 0.02 |
| Diabetes status | | |  |  |  |  |
|  | No | | Reference | - | Reference | - |
|  | Pre-existing type I/II diabetes | | 1.72 (0.92-3.19) | 0.09 | 2.11 (1.01-4.42) | 0.046 |
|  | Current gestational diabetes | | 0.90 (0.72-1.12) | 0.33 | 0.89 (0.69-1.13) | 0.35 |

## Supplementary Table 2. Univariate and multivariate logistic regression model of SGA by weight status with BMI>=30 from NSLHD 2013-2017 (n=2,466)

| Characteristics | | | Univariate model | | Multivariate model | |
| --- | --- | --- | --- | --- | --- | --- |
|  | | | OR (95% CI) | P Value | OR (95% CI) | P Value |
| BMI Obesity group | | |  |  |  |  |
|  | Class I Obesity (30-34.9) | | Reference | - | Reference | - |
|  | Class II obesity (35-39.9) | | 1.12 (0.70-1.81) | 0.63 | 1.65 (0.90-3.05) | 0.11 |
|  | Class III obesity (>=40) | | 0.66 (0.28-1.53) | 0.33 | 1.05 (0.37-2.98) | 0.93 |
| Age, y | | |  |  |  |  |
|  | <25 | | Reference | - | Reference | - |
|  | 25-29 | | 1.43 (0.54-3.80) | 0.47 | 0.73 (0.21-2.47) | 0.61 |
|  | 30-34 | | 1.12 (0.43-2.92) | 0.82 | 0.81 (0.27-2.44) | 0.71 |
|  | 35-39 | | 1.50 (0.57-3.94) | 0.41 | 1.20 (0.40-3.55) | 0.75 |
|  | 40+ | | 1.16 (0.36-3.73) | 0.80 | 0.67 (0.15-3.04) | 0.61 |
| Country of birth | | |  |  |  |  |
|  | | Anglosphere | Reference | - | Reference | - |
|  | | Asian | 2.64 (1.64-4.28) | <0.001 | 3.72 (1.93-7.18) | <0.001 |
|  | | Other | 1.87 (1.06-3.31) | 0.03 | 2.67 (1.30-5.51) | <0.01 |
| Term pregnancy | | |  |  |  |  |
|  | 0 | | Reference | - | Reference | - |
|  | 1 | | 0.38 (0.22-0.64) | <0.001 | 0.43 (0.24-0.77) | <0.01 |
|  | 2 | | 0.19 (0.07-0.53) | <0.01 | 0.26 (0.09-0.77) | 0.02 |
|  | 3+ | | 0.17 (0.04-0.73) | 0.02 | 0.20 (0.04-0.92) | 0.04 |
| Hypertension | | |  |  |  |  |
|  | No | | Reference |  |  |  |
|  | Essential HTN | | 2.76 (0.97-7.89) | 0.06 | 3.66 (0.99-13.50) | 0.05 |
|  | Gestational HTN | | 2.68 (1.34-5.34) | <0.01 | 3.85 (1.69-8.76) | <0.01 |
|  | Pre-eclampsia | | 10.62 (6.05-18.63) | <0.001 | 15.97 (7.80-32.68) | <0.001 |

## Supplementary Table 3. Univariate and multivariate logistic regression model of neonatal hypoglycaemia by obesity class.

| Characteristics | | | Univariate model | | Multivariate model | |
| --- | --- | --- | --- | --- | --- | --- |
|  | | | OR (95% CI) | P Value | OR (95% CI) | P Value |
| BMI Obesity group | | |  |  |  |  |
|  | Class I Obesity (30-34.9) | | Reference | - | Reference | - |
|  | Class II obesity (35-39.9) | | 0.88 (0.60-1.30) | 0.53 | 0.69 (0.43-1.12) | 0.14 |
|  | Class III obesity (>=40) | | 1.46 (0.92-2.32) | 0.11 | 1.33 (0.76-2.33) | 0.31 |
| Age, y | | |  |  |  |  |
|  | <25 | | Reference | - | Reference | - |
|  | 25-29 | | 1.90 (0.79-4.57) | 0.15 | 1.11 (0.36-3.42) | 0.85 |
|  | 30-34 | | 1.92 (0.82-4.52) | 0.13 | 0.87 (0.28-2.68) | 0.81 |
|  | 35-39 | | 2.61 (1.11-6.17) | 0.03 | 1.26 (0.41-3.90) | 0.69 |
|  | 40+ | | 3.12 (1.23-7.92) | 0.02 | 1.21 (0.36-4.05) | 0.76 |
| Country of birth | | |  |  |  |  |
|  | | Anglosphere | Reference | - | Reference | - |
|  | | Asian | 1.82 (1.26-2.65) | <0.01 | 1.14 (0.70-1.84) | 0.60 |
|  | | Other | 0.88 (0.53-1.47) | 0.64 | 0.96 (0.53-1.74) | 0.89 |
| Term pregnancy | | |  |  |  |  |
|  | 0 | | Reference | - | Reference | - |
|  | 1 | | 0.73 (0.50-1.07) | 0.11 | 0.78 (0.51-1.20) | 0.26 |
|  | 2 | | 0.62 (0.36-1.08) | 0.09 | 0.65 (0.36-1.16) | 0.14 |
|  | 3+ | | 0.67 (0.34-1.31) | 0.24 | 0.73 (0.35-1.54) | 0.41 |
| Diabetes status | | |  |  |  |  |
|  | No | | Reference | - | Reference | - |
|  | Pre-existing type I/II diabetes | | 23.03 (11.89-44.63) | <0.001 | 24.95 (10.92-57.01) | <0.001 |
|  | Current gestational diabetes | | 8.09 (5.77-11.34) | <0.001 | 7.87 (5.30-11.69) | <0.001 |

## Supplementary Table 4. Univariate and multivariate logistic regression model of birth timing by obesity class.

| Characteristics | | | Univariate model | | Multivariate model | |
| --- | --- | --- | --- | --- | --- | --- |
|  | | | OR (95% CI) | P Value | OR (95% CI) | P Value |
| BMI Obesity group | | |  |  |  |  |
|  | Class I Obesity (30-34.9) | | Reference | - | Reference | - |
|  | Class II obesity (35-39.9) | | 1.18 (0.83-1.66) | 0.36 | 1.16 (0.77-1.77) | 0.47 |
|  | Class III obesity (>=40) | | 1.20 (0.74-1.94) | 0.46 | 0.89 (0.46-1.74) | 0.74 |
| Age, y | | |  |  |  |  |
|  | <25 | | Reference | - | Reference | - |
|  | 25-29 | | 0.70 (0.39-1.24) | 0.22 | 0.54 (0.26-1.13) | 0.10 |
|  | 30-34 | | 0.74 (0.43-1.28) | 0.28 | 0.73 (0.37-1.43) | 0.36 |
|  | 35-39 | | 0.57 (0.32-1.01) | 0.055 | 0.50 (0.24-1.02) | 0.06 |
|  | 40+ | | 0.86 (0.43-1.69) | 0.66 | 0.86 (0.0.37-1.97) | 0.72 |
| Country of birth | | |  |  |  |  |
|  | | Anglosphere | Reference | - | Reference | - |
|  | | Asian | 0.92 (0.60-1.42) | 0.71 | 0.98 (0.57-1.67) | 0.93 |
|  | | Other | 1.10 (0.71-1.70) | 0.66 | 1.24 (0.76-2.04) | 0.39 |
| Term pregnancy | | |  |  |  |  |
|  | 0 | | Reference | - | Reference | - |
|  | 1 | | 0.55 (0.38-0.79) | <0.01 | 0.69 (0.46-1.03) | 0.07 |
|  | 2 | | 0.71 (0.43-1.15) | 0.16 | 0.96 (0.57-1.61) | 0.86 |
|  | 3+ | | 0.45 (0.22-0.93) | 0.03 | 0.50 (0.22-1.15) | 0.10 |
| Diabetes status | | |  |  |  |  |
|  | No | | Reference | - | Reference | - |
|  | Pre-existing type I/II diabetes | | 2.81 (1.28-6.17) | 0.01 | 3.20 (1.10-9.28) | 0.03 |
|  | Current gestational diabetes | | 1.26 (0.90-1.76) | 0.18 | 1.21 (0.80-1.82) | 0.38 |
| Hypertension | | |  |  |  |  |
|  | No | | Reference | - | Reference | - |
|  | Essential HTN | | 1.38 (0.54-3.52) | 0.50 | 1.05 (0.30-3.69) | 0.94 |
|  | Gestational HTN | | 1.16 (0.61-2.20) | 0.64 | 1.28 (0.59-2.76) | 0.53 |
|  | Pre-eclampsia | | 9.48 (5.96-15.06) | <0.001 | 9.04 (4.83-16.93) | <0.001 |

## Supplementary Table 5. Univariate and multivariate logistic regression model of birth defect by weight status with BMI>=30 from NSLHD 2013-2017 (n=2,466)

| Characteristics | | | Univariate model | | Multivariate model | |
| --- | --- | --- | --- | --- | --- | --- |
|  | | | OR (95% CI) | P Value | OR (95% CI) | P Value |
| BMI Obesity group | | |  |  |  |  |
|  | Class I Obesity (30-34.9) | | Reference | - | Reference | - |
|  | Class II obesity (35-39.9) | | 0.67 (0.33-1.39) | 0.29 | 0.79 (0.34-1.80) | 0.57 |
|  | Class III obesity (>=40) | | 2.21 (1.15-4.27) | 0.02 | 1.84 (0.82-4.15) | 0.14 |
| Age, y | | |  |  |  |  |
|  | <25 | | Reference | - | Reference | - |
|  | 25-29 | | 0.99 (0.32-3.05) | 0.99 | 3.19 (0.40-25.56) | 0.28 |
|  | 30-34 | | 0.96 (0.33-2.82) | 0.94 | 2.87 (0.37-22.05) | 0.31 |
|  | 35-39 | | 1.15 (0.38-3.46) | 0.80 | 4.81 (0.62-37.57) | 0.13 |
|  | 40+ | | 1.03 (0.27-3.91) | 0.96 | 3.64 (0.41-32.16) | 0.25 |
| Country of birth | | |  |  |  |  |
|  | | Anglosphere | Reference | - | Reference | - |
|  | | Asian | 0.70 (0.31-1.54) | 0.37 | 0.96 (0.41-2.25) | 0.92 |
|  | | Other | 0.23 (0.06-0.95) | 0.04 | 0.31 (0.08-1.33) | 0.12 |
| Term pregnancy | | |  |  |  |  |
|  | 0 | | Reference | - | Reference | - |
|  | 1 | | 0.34 (0.18-0.67) | <0.01 | 0.32 (0.16-0.64) | <0.01 |
|  | 2 | | 0.60 (0.27-1.36) | 0.23 | 0.52 (0.23-1.21) | 0.13 |
|  | 3+ | | 0.55 (0.19-1.62) | 0.28 | 0.35 (0.11-1.18) | 0.09 |
| Diabetes status | | |  |  |  |  |
|  | No | | Reference | - | Reference | - |
|  | Pre-existing type I/II diabetes | | 2.93 (0.88-9.83) | 0.08 | 2.92 (0.64-13.37) | 0.17 |
|  | Current gestational diabetes | | 0.95 (0.51-1.77) | 0.86 | 1.10 (0.54-2.21) | 0.80 |
| Hypertension | | |  |  |  |  |
|  | No | | Reference | - | Reference | - |
|  | Essential HTN | | 1.42 (0.34-5.98) | 0.63 | 0.66 (0.09-4.72) | 0.68 |
|  | Gestational HTN | | 0.54 (0.13-2.23) | 0.40 | 0.31 (0.04-2.27) | 0.25 |
|  | Pre-eclampsia | | 0.89 (0.21-3.71) | 0.87 | 0.58 (0.07-4.59) | 0.61 |

## Supplementary Table 6: Univariate and multivariate logistic regression model of caesarean section by obesity class

| Characteristics | | | Univariate model | | Multivariate model | |
| --- | --- | --- | --- | --- | --- | --- |
|  | | | OR (95% CI) | P Value | OR (95% CI) | P Value |
| BMI Obesity group | | |  |  |  |  |
|  | Class I Obesity (30-34.9) | | Reference | - | Reference | - |
|  | Class II obesity (35-39.9) | | 1.24 (1.02-1.51) | 0.03 | 1.36 (1.08-1.72) | <0.01 |
|  | Class III obesity (>=40) | | 1.72 (1.30-2.27) | <0.001 | 1.92 (1.37-2.70) | <0.001 |
| Age, y | | |  |  |  |  |
|  | <25 | | Reference | - | Reference | - |
|  | 25-29 | | 0.88 (0.61-1.27) | 0.49 | 0.84 (0.53-1.34) | 0.47 |
|  | 30-34 | | 1.38 (0.97-1.96) | 0.07 | 1.43 (0.91-2.23) | 0.12 |
|  | 35-39 | | 1.74 (1.21-2.49) | <0.01 | 1.78 (1.13-2.82) | 0.01 |
|  | 40+ | | 1.96 (1.28-3.01) | <0.01 | 2.10 (1.24-3.55) | <0.01 |
| Country of birth | | |  |  |  |  |
|  | | Anglosphere | Reference | - | Reference | - |
|  | | Asian | 1.13 (0.90-1.43) | 0.29 | 1.25 (0.93-1.67) | 0.14 |
|  | | Other | 1.37 (1.07-1.76) | 0.01 | 1.21 (0.91-1.62) | 0.20 |
| Term pregnancy | | |  |  |  |  |
|  | 0 | | Reference | - | Reference | - |
|  | 1 | | 0.89 (0.72-1.11) | 0.30 | 0.88 (0.70-1.11) | 0.28 |
|  | 2 | | 0.79 (0.59-1.05) | 0.10 | 0.75 (0.55-1.01) | 0.058 |
|  | 3+ | | 0.63 (0.44-0.91) | 0.01 | 0.53 (0.36 -0.78) | <0.001 |
| Diabetes status | | |  |  |  |  |
|  | No | | Reference | - | Reference | - |
|  | Pre-existing type I/II diabetes | | 4.68 (2.29-9.55) | <0.001 | 3.37 (1.43-7.93) | <0.01 |
|  | Current gestational diabetes | | 1.13 (0.93-1.36) | 0.23 | 0.90 (0.72-1.13) | 0.38 |
| Hypertension | | |  |  |  |  |
|  | No | | Reference |  |  |  |
|  | Essential HTN | | 2.76 (0.97-7.89) | 0.058 | 1.23 (0.64-2.36) | 0.53 |
|  | Gestational HTN | | 2.68 (1.34-5.34) | <0.01 | 0.93 (0.60-1.44) | 0.74 |
|  | Pre-eclampsia | | 10.62 (6.05-18.63) | <0.001 | 2.94 (1.58-5.46) | <0.01 |

## Supplementary Table 7: Univariate and multivariate logistic regression model of gestational diabetes by obesity class

| Characteristics | | | Univariate model | | Multivariate model | |
| --- | --- | --- | --- | --- | --- | --- |
|  | | | OR (95% CI) | P Value | OR (95% CI) | P Value |
| BMI Obesity group | | |  |  |  |  |
|  | Class I Obesity (30-34.9) | | Reference | - | Reference | - |
|  | Class II obesity (35-39.9) | | 1.21 (0.96-1.52) | 0.11 | 1.46 (1.12-1.92) | <0.01 |
|  | Class III obesity (>=40) | | 1.49 (1.09-2.04) | 0.01 | 1.66 (1.14-2.41) | <0.01 |
| Age, y | | |  |  |  |  |
|  | <25 | | Reference | - | Reference | - |
|  | 25-29 | | 1.89 (1.08-3.32) | 0.03 | 2.04 (0.96-4.37) | 0.06 |
|  | 30-34 | | 2.47 (1.44-4.25) | <0.01 | 2.56 (1.23-5.33) | 0.01 |
|  | 35-39 | | 3.70 (2.15-6.38) | <0.001 | 3.99 (1.91-8.33) | <0.001 |
|  | 40+ | | 4.58 (2.51-8.34) | <0.001 | 4.95 (2.26-10.88) | <0.001 |
| Country of birth | | |  |  |  |  |
|  | | Anglosphere | Reference | - | Reference | - |
|  | | Asian | 2.49 (1.94-3.20) | <0.001 | 3.00 (2.22-4.06) | <0.001 |
|  | | Other | 1.16 (0.86-1.57) | 0.33 | 0.99 (0.69-1.41) | 0.95 |
| Term pregnancy | | |  |  |  |  |
|  | 0 | | Reference | - | Reference | - |
|  | 1 | | 0.88 (0.68-1.13) | 0.30 | 0.81 (0.62-1.05) | 0.12 |
|  | 2 | | 1.01 (0.73-1.41) | 0.94 | 0.94 (0.67-1.34) | 0.75 |
|  | 3+ | | 0.79 (0.51-1.22) | 0.29 | 0.71 (0.46 -1.11) | 0.14 |
| Hypertension | | |  |  |  |  |
|  | No | | Reference |  |  |  |
|  | Essential HTN | | 1.07 (0.56-2.06) | 0.84 | 0.73 (0.32-1.65) | 0.45 |
|  | Gestational HTN | | 1.36 (0.92-2.01) | 0.12 | 1.37 (0.86-2.18) | 0.19 |
|  | Pre-eclampsia | | 1.34 (0.81-2.22) | 0.25 | 1.02 (0.50-2.09) | 0.95 |

## Supplementary Table 8: Univariate and multivariate logistic regression model of preeclampsia toxemia by obesity class

| Characteristics | | | Univariate model | | Multivariate model | |
| --- | --- | --- | --- | --- | --- | --- |
|  | | | OR (95% CI) | P Value | OR (95% CI) | P Value |
| BMI Obesity group | | |  |  |  |  |
|  | Class I Obesity (30-34.9) | | Reference | - | Reference | - |
|  | Class II obesity (35-39.9) | | 1.59 (0.96-2.64) | 0.07 | 1.53 (0.80-2.92) | 0.20 |
|  | Class III obesity (>=40) | | 2.46 (1.33-4.55) | <0.01 | 1.40 (0.55-3.56) | 0.48 |
| Age, y | | |  |  |  |  |
|  | <25 | | Reference | - | Reference | - |
|  | 25-29 | | 0.61 (0.28-1.32) | 0.21 | 0.76 (0.23-2.58) | 0.67 |
|  | 30-34 | | 0.55 (0.26-1.15) | 0.11 | 0.85 (0.26-2.70) | 0.78 |
|  | 35-39 | | 0.48 (0.22-1.06) | 0.07 | 0.98 (0.28-3.36) | 0.97 |
|  | 40+ | | 0.44 (0.15-1.33) | 0.15 | 0.63 (0.14-2.89) | 0.55 |
| Country of birth | | |  |  |  |  |
|  | | Anglosphere | Reference | - | Reference | - |
|  | | Asian | 0.87 (0.46-1.67) | 0.68 | 0.57 (0.20-1.67) | 0.31 |
|  | | Other | 0.65 (0.30-1.43) | 0.29 | 0.88 (0.33-2.33) | 0.80 |
| Term pregnancy | | |  |  |  |  |
|  | 0 | | Reference | - | Reference | - |
|  | 1 | | 0.37 (0.20-0.69) | <0.01 | 0.37 (0.19-0.69) | <0.01 |
|  | 2 | | 0.13 (0.03-0.55) | <0.01 | 0.12 (0.03-0.54) | <0.01 |
|  | 3+ | | 0.37 (0.11-1.25) | 0.11 | 0.34 (0.10 -1.15) | 0.08 |
| Diabetes status | | |  |  |  |  |
|  | No | | Reference |  |  |  |
|  | Pre-existing type I/II diabetes | | 3.49 (1.20-10.18) | 0.02 | 1.40 (0.20-9.59) | 0.73 |
|  | Current gestational diabetes | | 1.34 (0.81-2.21) | 0.26 | 1.00 (0.49-2.04) | 0.99 |
